# Effect of different concentrations of growth regulator on callus induction of the rice lineage AB11047

**DOI:** 10.1186/1753-6561-8-S4-P118

**Published:** 2014-10-01

**Authors:** Natália Segatto, Martina Bianca Fuhrmann, Tatiane Casarin, Carla Ferreira Silveira, Luciana Bicca Dode, Luciano Silva Pinto, Ariano Martins De Magalhães Júnior

**Affiliations:** 1 Cdtec-Ufpel, Pelotas, Brazil; 2 Embrapa Clima Temperado, Pelotas, Brazil

## Background

The lineage AB11047(*Oryza sativa *L.), developed by the rice breeding program of Embrapa, has a 126-day biological cycle, large grains with high ratio starch/husk and high productivity. It is resistant to the main rice diseases and degrain. Produces an average weight of 52g, while the majority of irrigated rice cultivars presents an average weight of 25g. This lineage represents a new source to ethanol production and/or animal feed, since the size of the grain is twice as big as the regular one, and it can be used for human consumption. It is observed that both economic and social demands of the rice sector, as the ethanol production chain indicate that there is an urgent need for cultivars that meet the agro-energy market.

*In vitro *vegetal tissue cultivation is an important tool in the search for the expansion of plant genetic variability, being essential to all kinds of plant breeding programs. An efficient plant regeneration protocol is necessary for the plant genetic transformation success. In the rice cultivation case, the process can be initiated by callus induction from mature seeds, which will directly interfere in the regeneration success, due to differences in the regenerative potential between different cultivars. This process is a prerequisite for the success of rice biotech [1]. In theory, every seed that can germinate can be used, however, the response of seeds to callus induction is highly dependent on the genotype of rice [2].

This study aimed to establish the optimal concentration of the growth regulator 2,4 dichlorophenoxyacetic acid (2,4D) to promote potentially organogenic callus from lineage A11047 mature seeds.

## Methods

Rice seeds were manually dehusked and disinfected by immersion in solution of 70% alcohol (v/v) under slightly agitation for 2 minutes, followed by rinse with sterile distilled water, and immersion in solution of sodium hypochlorite 3% (v/v) under slightly agitation for 25 minutes. The Murashige & Skoong (MS) medium plus 3% (w/v) of sucrose, 7 g.L^-1 ^of Agar and different concentrations of growth regulator 2,4D was used. The treatments consisted in a control group, without the addiction of the growth regulator; treatment 1, with 2 mg.L^-1 ^of 2,4D; treatment 2, 2,5 mg.L^-1 ^of 2,4D; and treatment 3, 3 mg.L^-1 ^of 2,4D. The cultivars were in the incubator BOD at 28ºC, in the dark.

The experimental delineation was completely randomized, knowing that for each genotype were used 8 repetitions, with 10 seeds each. After 7 days, the frequency of the callus formation was assessed. The data underwent variance analysis and the averages compared by the Scott-Knott's test, at 5% probability.

## Results and conclusions

A significant difference between the obtained averages in the different concentrations of 2,4D used and the treatment control was observed. The treatments T1 and T2 showed an average frequency of 60% and 61,2% of callus, respectively, with no statistic difference between them. The T3 was lower than T1 and T2 showing 46,2% of callus. The treatments T1 and T2 proved to be the most appropriate ones to the induction of callus from the lineage A11047.
